# Sleep and nutritional profile of endurance and ultra-endurance running athletes

**DOI:** 10.5935/1984-0063.20220076

**Published:** 2022

**Authors:** Nadia Esteves dos Santos, Natalia Vilela Silva Daniel, Beatriz Franco, Andre Marana Bastos, Taisa Belli, Andrea Maculano Esteves

**Affiliations:** 1 Universidade Estadual de Campinas, Faculdade de Ciências Aplicadas - Limeira - São Paulo - Brazil; 2 Universidade Estadual de Campinas, Faculdade de Educação Física - Campinas - São Paulo - Brazil

**Keywords:** Running, Athletes, Sleep, Nutrition Personnel

## Abstract

Sleeping and eating before and during an ultramarathon can directly affect an athlete’s performance, who may also have their physiological adaptations and recovery process hindered by sleeping problems. Endurance and ultra-endurance athletes may have different sleep and nutrition profiles. Thus, this study aimed to describe the sleep profile (during preparation) and nutritional profile (during competition) of endurance (10-20km) and ultra-endurance (50-100km) running athletes. For this, 16 healthy volunteers answered questionnaires related to sleep quality (Pittsburgh sleep quality index), chronotype (morningness-eveningness questionnaire), and sleepiness (excessive daytime sleepiness questionnaire). Immediately after a competition, a form prepared by the research team about nutritional variables and volunteers’ food records during the competition was applied. According to test scoring criteria (Pittsburgh sleep quality index >5; sleepiness >10), endurance running athletes showed low sleep quality. In addition, all athletes showed consumption of carbohydrates and lipids below the recommended, but excessive consumption of proteins. A positive association between sleepiness and sodium intake in endurance runners was observed (r=0.862; *p*=0.027). Sleep efficiency and race time showed a negative correlation only for ultra-endurance athletes (r=-0.834; *p*=0.039). The data obtained show that endurance athletes presented more sleep pattern alterations, however, endurance and ultra-endurance athletes showed inadequate nutritional consumption during the competition.

## INTRODUCTION

Endurance races are formed by routes that gather short distances (5km to 40km). Ultramarathons, in turn, are running events with a course higher than a marathon, with distances from 50km to 160km, being held on various courses (e.g., track and field, trails, mountain coasts, and deserts) and presented as single or multiple stage events^1^.

Over the past few years, scientific research has focused on understanding the set of physiological and pathophysiological adjustments resulting from competitions of this nature. In this sense, appropriate food intake and healthy sleep habits are among the factors that influence both the athletes’ health and performance^2-4^.

Inadequate pre-race sleep habits and strategies can potentially exacerbate fatigue, increase the risk of injury, hallucinations, and lead athletes to withdraw from the competition. Even when they manage their sleep, with short naps for example, there is little evidence to indicate how this should be accomplished during prolonged athletic performance^5^. Given this, sleep is a subject that lacks in the scientific literature focused on evidence of resistance^3^.

Acute or chronic sleep deprivation is associated with increased sleepiness and reduced cognitive functioning, particularly attention, which potentially leads to reduced performance in resistance events^6-8^. Sleep deprivation leads to increased injuries, reduced muscle glycogen stores and, consequently, alters muscle recovery. These changes affect different aspects of performance^3,9,10^. Therefore, the athlete needs adequate preparation so that on the day of the competition the consequences are as small as possible.

Aspects related to food logistics and food consumption during ultramarathons are also a relevant theme, since the ultramarathoner presents great energy need and multiple barriers to adequate food intake, such as gastrointestinal discomfort, topographic and environmental conditions. Recent studies have proposed guidelines for nutrition, evidencing the need for future research^3,11,12^.

In the study of Silva et al. (2022)^13^, when evaluating an ultramarathon athlete, they observed that the nutritional intake was similar to that of other triathletes who participated in competitions with an average duration of 12h^14^. The authors also noted that the athlete presented energy, carbohydrate, and protein intake lower than recommended by the guidelines for ultra-endurance sports^11^.

Although the literature is extensive on nutritional recommendations^15^ and association of sleep and performance for endurance sports^16^, little is known about these aspects when the scenario is ultra-endurance sports. Nutritional recommendations for long-distance sports have been published recently^11-12^, but as there are still no specific sleep guidelines for ultra-endurance athletes, we considered results from studies on endurance athletes.

Considering that runners do not usually sleep during ultramarathons, especially at events lasting up to one night^2^, our hypothesis is that pre-competition sleep strategies are specially designed to overcome sleep deprivation during the race. This challenge is not faced by endurance athletes, who will not spend more than 24 hours running and, therefore, will not necessarily be sleep-deprived during exercise. Considering that the physiological demands are different in endurance and ultra-endurance sports, it is important to understand whether sleep and eating patterns also differ in these two modalities.

Thus, the main objective of this study was to describe the sleep profile of athletes during preparation and their nutritional profile during endurance and ultra-endurance competition. In addition, it was possible to associate the athletes’ nutritional strategies, sleep pattern and performance in the competition.

## MATERIAL AND METHODS

### Subjects

A sample of 16 amateur athletes participated voluntarily (*M_age_* = 40.22±10.22; 7 men, 9 women). At the time of the data collection, all athletes participated in the Br135 Ultra Street Circuit (10-20km endurance n=7, and 50-100km ultra-endurance n=9) in the mountains of Serra da Mantiqueira/Brazil.

All procedures were approved by the research ethics committee of UNICAMP (4.179.685).

### Procedures

In the week preceding the competition, the volunteers answered online questionnaires about chronotype, sleep quality, and sleepiness.

The morningness-eveningness questionnaire (MEQ) characterizes the period of the day in which the individual has a greater tendency and willingness to perform his activities, which can be caused by a biological, physiological, and psychological phenomena. The questionnaire contains 19 multiple choice questions, and the total of the scores ranges from 16 to 86 points, being classified as extreme afternoon (16 to 33), moderate afternoon (34 to 44), indifferent (45 to 65), moderate morning (66 to 76) and extreme morning (77 to 86)^17^.

The Pittsburgh sleep quality index (PSQI) indicates the sleep quality index of volunteers. The questionnaire consists of 21 items that assess sleep quality and its disorders through the last month’s record of the following components: sleep latency, sleep duration, sleep efficiency, sleep disorders, medication use, and dysfunctions during the day. The classification criterion is based on the total score obtained, grouped according to the participants’ sleep, as good (below 4 points) or bad (equal to 5 or higher)^18^.

The excessive daytime sleepiness questionnaire contains 8 questions and assess the overall level of excessive daytime sleepiness at active and passive moments. The score goes from 0 (no chance) to 3 (high chance) for each question. Measurement values are obtained by summing all scores and qualified as: normal (0 to 6), limit (7 to 9), minimum sleepiness (10 to 14), moderate sleepiness (15 to 20), and severe sleepiness (above 20)^19^.

Anthropometric data were reported before the race, and body mass and height values were used to calculate and to classify the Body Mass Index (BMI)^20^.

To assess food consumption during the race, volunteers completed a form prepared by the research team, which recorded date, time, food, and their amounts in grams or liters through a material with photographies^21^. With these data, the total energy consumed during the race and the amounts of carbohydrates, lipids, proteins, and micronutrients were calculated using the Avanutri software, version 3.1.1. If the nutritional information was not available in the software, a food composition table^22^ and the label of the ingested products were consulted.

To calculate the energy requirement, we used the method of Harris and Benedict (1918)^23^, first defining the basal metabolism rate, and then applying the level of physical activity.

The training volume and sessions per week were reported by the athletes through online spreadsheets.

### Statistical analysis

The statistical analysis was performed using Statistic software (version 7.0, StatSoft Inc., Tulsa, OK, USA), with the data being expressed as mean and standard deviation (SD). The Shapiro-Wilk normality test was used for the normalization analysis. The Mann-Whitney test was used for non-normal parameters. Correlations were performed using the Pearson’s chi-squared test for normal parameters. The Hedge’s g statistic was used to measure the effect size (ES) for the difference between means. The values obtained were used to define the effect sizes as trivial (d<0.2); small (0.2<d<0.5); medium (0.5<d<0.8); and large (d>0.8)^24^. The critical level of significance was *p*<0.05.

## RESULTS

The study included 16 runners of both sexes: 7 endurance runners (10-20km) and 9 ultra-endurance runners (50-100km). The participants’ general characteristics are presented in Table 1. Although no significant differences were found, we observed that the ultra-endurance runners presented lower BMI (ES=1.04) and higher training volume (ES=1.14) than endurance runners.

**Table 1:** General characteristics of the participants.

Characteristics	Endurance (n=7)	Ultra-endurance (n=9)	p	Hedge’s g	Confidence interval 95%
Age (Years)	35.14 ± 12.32	45.30 ± 8.12	0.285	0.95	-0.04 - 1.94
Weight (kg)	67.30 ± 10.71	60.30 ± 6.60	0.123	0.77	-0.20 - 1.74
Height (meters)	1.66 ± 0.06	1.68 ± 0.05	0.485	0.35	-0.59 - 1.29
Body mass index (kg/m^2^)	24.37 ± 3.39	21.51 ± 1.77	0.050	1.04	0.04 - 2.04
Running practice (years)	5.42 ± 6.50	10.18 ± 11.40	0.285	0.47	-0.48 - 1.42
Sessions per week	3 ± 1.50	4 ± 2.50	0.363	0.44	-0.50 - 1.39
Training volume (km/week)	13.57 ± 12.45	62.2 ± 52.23	0.237	1.14	0.13 - 2.15

Notes: Values presented in mean (SD); Mann-Whitney test, *p*<0.05; CI: Confidence interval; Hedge’s g: trivial (d<0.2), small (0.2<d<0.5), medium (0.5<d<0.8), and large (d>0.8).

In Table 2, when analyzing the groups of runners according to sleep quality and sleepiness, the results showed that endurance athletes presented a PSQI, sleepiness, and sleep efficiency (borderline) worse than population reference values^25^. However, although no significant differences were found for sleep, we observed that the ultra-endurance athletes presented better PSQI (ES=0.55) and sleep latency (ES=0.55) than endurance. Endurance athletes also presented an excessive average of daytime sleepiness (with a score >10 in the Epworth questionnaire), while ultra-endurance athletes presented close values, but did not reach the established cutoff range.

**Table 2:** Descriptive of sleep quality (PSQI), sleepiness and chronotype.

Variables	Endurance (n=7)	Ultra-endurance (n=9)	p	Hedge’s g	Confidence interval 95%
PSQI	6.14 ± 2.74	4.66 ± 2.40	0.261	0.55	-0.40 - 1.50
Sleep efficiency (%)	85 ± 8.60	88 ± 16	0.670	0.21	-0.72 - 1.15
Sleep latency (min)	20.71 ± 14.50	13.88 ± 9.06	0.387	0.55	-0.40 - 1.50
Sleepiness	10.14 ± 3.90	9.55 ± 4.16	0.708	0.14	-0.80 - 1.07
Chronotype	53.71 ± 10.47	59.66 ± 7.76	0.426	0.62	-0.33 - 1.58

Notes: Values presented in mean (SD); Chronotype (Horne & Ostberg Questionnaire)^7^: extreme afternoon (16 to 33), moderate afternoon (34 to 44), indifferent (45 to 65), morning moderate (66 to 76) and extreme morning (77 to 86); Sleep quality (PSQI): Poor sleep (equal to 5 or higher); Sleep efficiency (adequate >85%); Latency: Poor sleep (>30 minutes); Sleepiness (Epworth questionnaire): >10. Mann-Whitney test, *p*<0.05; CI: Confidence interval; Hedge’s g: trivial (d<0.2), small (0.2<d<0.5), medium (0.5<d<0.8), and large (d>0.8).

**Figure 1:**
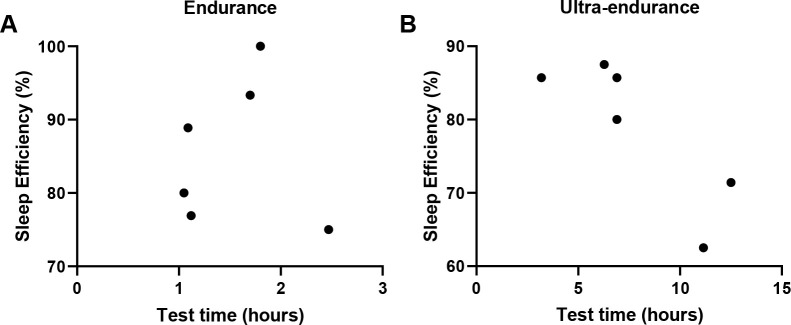
Correlation between sleep efficiency (%) and test time. **A.** Endurance (r=0.001; *p*=0.998); **B.** Ultra-endurance (r=-0.834; *p*=0.039), Pearson’s correlation test.

A negative correlation was found between sleep efficiency and the test time of ultra-endurance athletes in this competition (r=-0.834; *p*=0.039, Figure 1), but not for endurance athletes (r=0.001; *p*=0.998). For test time, sleep latency, sleepiness, and PSQI no correlation was observed for endurance and ultra-endurance.

Table 3 shows the results on the perception of nutritional variables for the competition period and water consumption. Of 16 participants, 43.75% answered that for a better performance in the running race, one should consume foods rich in simple carbohydrates during the competition, 37.50% said that one should consume foods rich in complex carbohydrates, and 18.75% believed the association between foods rich in protein and carbohydrates was the best option. As for liquids, the runners indicated water mixed with energy repositors as the main beverage for hydration (87.50%), followed by energy repositors (6.25%) and water only (6.25%).

**Table 3:** Perception of nutritional variables for the competition period and water consumption of competitors.

Nutritional variables	N	%
**1: For the best performance**		
A: Foods’s rich in simple carbohydrates	7	43.75
B: Foods’s rich in complex carbohydrates	6	37.50
C: Protein-rich foods	-	-
D: Lipid-rich foods	-	-
E: Foods’s rich in protein and carbohydrates	3	18.75
**2: Water consumption**		
A: No water consumption	-	-
B: Water consumption	1	6.25
C: Consumption of energy repositors	1	6.25
D: Water consumption and energy repositors	14	87.50

Table 4 presents data on the participants’ daily energy needs and the composition of macronutrients and micronutrients compared to the reference values^26^.

**Table 4:** Consumption of macronutrients and micronutrients during the athletes’ race of 10-20km and 50-100km.

TEST	Weight (kg)	TEN (Kcal)	Mean Consumption (Kcal)	CHO (g)	LIP (g)	PTN (g)	Food Fiber (g)	Sodium (mg)	Water (mL/L)
**Endurance (n=7)**	78	3149.82	1657.80	231.75#	78.30^a^	82.80^#^	7.40	1.383^a^	0
	60	3274.65	1723.50	251.20^#^	68.42^*^	78^*^	11.30	3.138	500
	75	3180.03	1673.70	231.73^#^	65.55^#^	78^*^	17.40	2.167^a^	1.100^a^
	78	2882.30	1517	84.58^#^	16.44^#^	93.60^a^	28.50^*^	1.948^a^	400
	69	3497.11	1840.90	175.47^#^	90.58^*^	72^#^	0	0	0
	65	2810.48	1479.20	129.93^#^	36.90^#^	90^*^	8.30	2.012^a^	500
	46	2471.14	1300.60	153.32^#^	26.30^#^	55.20^a^	5.10	1.150^a^	2.000^a^
**Mean**							11.14 (8.64)	1.685.42 (505.24)	642.85 (652.15)
**Ultra-end (n=9)**	59	1802.53	1.386.50	236^#^	91.83^*^	70.80^a^	12.10	3.407	2.800^a^
	72	1843.38	1.686.20	210.54^#^	75.10^*^	86.40^a^	23.10	2.945	2.000^a^
	61	2634.35	1.386.50	220.16^#^	66.41^*^	75.60^*^	35	2.427	1.150
	52	2651.83	1.395.70	280.40^#^	45.73^#^	73.20^*^	0	0	0
	63	2377.28	1.251.20	370.95^#^	20.80^#^	72^#^	19.90	1.823^a^	1.150^a^
	60	2536.88	1.335.20	263.86^#^	42.05^#^	82.80^*^	17.80	2.105^a^	4.000^a^
	69	2241.81	1.179.90	211.73^#^	52.93^#^	64.20^#^	20.20	3.515	1.800^a^
**Mean**							14.21 (11.52)	1.792 (1.374)	1.394 (1.314)

Notes: Values presented in Mean (SD); TEN = Total energy need; CHO = Carbohydrate; LIP = Lipid; PTN = Protein; #below;

* above and

a ideal.

When the specific indication was observed for athletes regarding the energy intake, we also observed that all athletes consumed fewer calories than the energy demand estimated during physical activity. Regarding macronutrients intake, the consumption was compared to literature recommendations, which are 1.2g/kg/day of protein; 1.0g/kg/day of fats, and 8g/kg/day of carbohydrates. As to carbohydrate consumption, 100% consumed amounts lower than recommended. Regarding lipids, 35.72% had higher intake than recommended, 7.14% had ideal intake, and 57.14% had lower intake than recommended. As for proteins, 28.60% had ideal intake, 28.57% had lower intake, and 42.85% had higher intake than recommended^26^.

Regarding fiber consumption, the athletes’ ingestion was below the daily dietary fiber intake recommendations for healthy adults, which suggest 38g for men and 25g for women of the studied age group^27^. As for sodium, the recommendation for healthy adults, according to the dietary reference intakes^27^, is 1,500mg of sodium, and we can observe that only four athletes had a sodium intake lower than that. For water consumption, 43.75% had adequate fluid consumption during the test.

**Figure 2:**
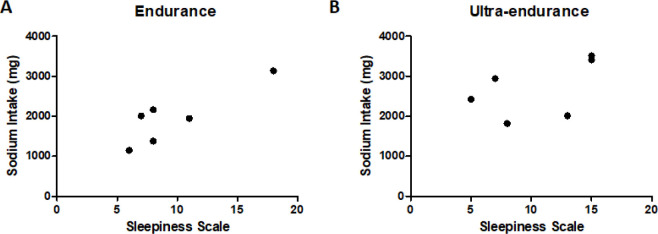
Correlation between sleepiness and sodium intake. **A.** Endurance (r=0.862; *p*=0.027); **B.** Ultra-endurance (r=0.512; *p*=0.299), Pearson’s correlation test.

A significant correlation was found between sleepiness and sodium intake (mg) in the endurance (r=0.8629; *p*=0.02). However, it has not been observed for ultra-endurance athletes (Figure 2).

## DISCUSSION

This study evaluated the sleep pattern of endurance and ultra-endurance running athletes and their nutritional profile during the competition. Among the main findings we can highlight the poor sleep quality (by PSQI) of running athletes, in addition to changes in sleepiness and sleep efficiency (borderline), when compared with the reference values found in the literature^25^, as well as a worse PSQI (ES=0.55) and sleep latency (ES=0.55) than ultra-endurance athletes. When nutritional strategies are compared with guidelines for endurance sports, we can note that most athletes had insufficient energy and carbohydrate; protein had a variation tending towards excessive, and fat intake.

In our results, we found a daytime sleepiness level of 43.75% among athletes, corroborating the study of Martin et al. (2018)^3^, which investigated sleep habits and strategies of ultramarathoners. In the sample studied, 37.6% had a higher sleepiness score than the classic cut-off score of 10, reflecting excessive daytime sleepiness.

We found a negative correlation in sleep efficiency and running time in 100km athletes. Brager et al. (2020)^28^, observed that 100 miles runners have 2 times more sleep disorders, prolonged mid-night arousal, restlessness and night sweats compared to 50 miles runners, directly interfering in sleep quality and efficiency, which may influence the ultramarathon performance.

The training frequency/volume can influence performance. Our study presented a weekly volume of 62.2km for ultra-endurance and 13.57km for endurance athletes, with a remarkable difference between training volumes. Furthermore, the trained group (ultra-endurance) frequency corresponded to 4±2.50 sessions per week, and the moderately trained group (endurance), 3±1.50 sessions per week. These results corroborate with those of Dal Pupo et al. (2011)^29^ in which national-level runners presented a volume from 40 to 70km per week, being compatible with those found in this research.

A personalized diet approach in ultra-endurance races, including the intake of important nutrients and energy compatible with the high demand of the exercise, is needed to improve performance efficiency during the test^2^. The difficulty of having an energy consumption similar to the caloric expenditure of the activity not only was observed in this study but has been described in the literature as something frequently observed in ultramarathons^2^.

During exercise, there are significant losses of liquids and minerals, as well as an increase in the sweating rate, ranging from 1 to 2 liters of liquids per hour of intense activity, so adequate hydration is essential to maintain physical performance and health^4^. When we looked at our results regarding mileage close to a marathon, only two participants of the 10-20km group had adequate fluid consumption during the running.

A study by Hoffman et al. (2013)^30^ found that the amount of sodium consumed during an ultramarathon is not associated with performance. However, the increase in the consumption of foods rich in sodium may encourage ultra-runners to establish more adequate hydration behaviors during events. A diet rich in sodium is associated with poor sleep quality, as it impairs circadian rhythms, causing sleep fragmentation, short sleep duration, increased awakenings and daytime sleepiness^31^. In our results, we found that endurance athletes had daytime sleepiness associated with sodium intake, as well as the sleep alterations described above.

The balance and nutritional pattern include the consumption of dietary fiber in the 50g/day diet as a positive reducing adipose tissue, increasing runners’ aerobic adaptation and physical performance^32^. Our findings showed that the fiber ingestion of athletes was below the daily dietary fiber intake recommended for healthy adults^27^. But considering that we evaluated nutritional consumption during an endurance/ultra-endurance event, it is possible that they avoided the intake of fiber during the race to minimize risks of gastrointestinal discomfort while running, as recommended by the literature^2,15^.

Ensuring that lipid intake is not excessively low is extremely important for the maintenance of good health and sports performance, since studies already show negative effects on the health of individuals who consume less than 20% of this macronutrient^15^. In the study, 35.72% had higher intake than recommended, 7.14% had ideal intake, and 57.14% had lower intake than recommended by the American College of Sports Medicine^15^.

Both endurance and ultra-endurance athletes had inadequate nutritional intake, and most athletes had a nutritional intake lower or higher than recommended for endurance exercises. Note that, inadequate nutritional intake during sports practice can compromise the athlete’s performance^15^; therefore, we emphasize the need to raise the athletes’ awareness so that they have a food consumption in line with the recommendations. Costa et al. (2019)^12^ highlights that more studies are warranted in this area of ultra-endurance sports, and that other authors need to evaluate nutritional strategies of successful ultramarathon runners in order to compare them with standards and recommendations for endurance exercise. This study may contribute to the literature by describing the dietary pattern of both endurance and ultra-endurance athletes, who participated in the same competitive event, with the same topographical and climatic conditions.

When comparing the results of endurance and ultra-endurance athletes, we observed that endurance athletes had worse sleep quality (PSQI) and higher sleep latency. These same athletes also presented a sleep latency considered excessive, and all the data suggest inadequate sleep pattern. One possible explanation is that ultra-endurance athletes, for facing sleep deprivation during an ultramarathon, are more concerned about having better sleep habits during the usual training period (or before competition), in an attempt to minimize the damage caused by the lack of sleep during the competition. Although endurance athletes do not face sleep deprivation during competition, they should be aware of the negative effects of inadequate sleep on sports performance.

In this context, the obtained data show that the athletes presented alterations in sleep pattern on preparation days and inadequate nutritional consumption during the competition, due to the nutritional complexity of the event, probably influencing their performance. As a limitation of the study, it was not possible to collect the general physiological requirements for completing an ultra-resistance race.

Therefore, studies with interventions are necessary to improve these parameters, which can interfere not only in the performance, but also in the athlete’s quality of life.
